# Braving the New World

**DOI:** 10.1371/journal.ppat.1005417

**Published:** 2016-04-28

**Authors:** Lindsey M. Hutt-Fletcher

**Affiliations:** Department of Microbiology and Immunology, Center for Molecular and Tumor Virology and Feist-Weiller Cancer Center, Louisiana State University Health Sciences Center, Shreveport, Louisiana, United States of America; University of Florida, UNITED STATES

I came to the United States in 1973 because the then Chair of Bacteriology and Immunology at the University of North Carolina at Chapel Hill (UNC), the late Philip Manire, did a sabbatical at the Lister Institute in London where I was working on my Ph.D. “What are you going to do when you finish?” he asked. I was one of those students that my current Chair berates for not having thought about life after graduation and had to confess that I didn’t really know. I had been working as a research assistant and had more or less assumed that I would keep doing the same thing; no one had suggested anything to the contrary. “Why not come to the States for two years and see what it is like?” he said. “There is someone in my department working on this new virus, Epstein–Barr virus [EBV], maybe you can work with him.” It seemed like a good idea, and 42 years later, I am just closing a career working on that “new virus,” EBV, and I am still in the US.

EBV appealed to me because I liked immunology, I thought viruses were really interesting, and a virus that infected the immune system seemed to represent an ideal combination for study. In Joe Pagano’s lab at UNC, I had pretty much free range in what I did. I was in a supportive environment with wonderful colleagues in what eventually became the Lineberger Comprehensive Cancer Center. Looking back, it is amazing to me how, even as a very junior postdoc, busy, established investigators would take time to talk with me and discuss ideas. At UNC, I was also encouraged to write grants and got my first chance to fly independently.

That new—actually quite old—virus, EBV, infects just about all of us. We carry it in our B lymphocytes and in our epithelial cells, and generally speaking, it does not seem to do most of us much harm. If we are first infected after the age of about ten or 11, there is a good chance that we will get infectious mononucleosis—temporarily debilitating, but only rarely any worse. A bout of infectious mononucleosis does, however, slightly increase the chance of subsequently developing Hodgkin’s lymphoma or multiple sclerosis, and as we age, an enormous part of our immune system is devoted to keeping EBV in check, probably reducing our ability to respond to other pathogens. If you lose immune control of the virus, B cell lymphomas can develop, and such EBV-driven tumors are a scourge of transplant recipients and AIDS patients. More difficult to explain, and the subject of considerable research effort over the years, has been the association of EBV with nasopharyngeal carcinomas, a subset of gastric cancers, and a variety of rarer cancers such as natural killer/T cell lymphomas and leiomyosarcomas. It has been estimated that EBV is responsible for about 200,000 new cases of cancer worldwide, every year.

As my own research on EBV continued, my lab began to focus on the envelope proteins of the virus that allow the virus to attach to a cell, fuse its envelope with the cell membrane, and get inside. After all, no virus can survive or cause disease, including cancer, unless it can penetrate a cell. So we studied virus proteins involved in attachment and entry into its two major targets, B lymphocytes and epithelial cells, and we sought to identify partners on the cell surface that collaborated with the virus. Over the years, we discovered that the virus uses HLA class II to trigger its entry into a B cell and a subset of alpha v integrins to trigger its entry into epithelial cells. We found that a complex of three virus proteins, called gH, gL, and gp42, regulates B cell fusion and that a subset of only two of them, gH and gL, regulates epithelial cell fusion. To our delight, this allowed us to uncover a mechanism that the virus uses to switch its tropism alternately from B cell to epithelial cell and back again to maintain a cycle of low level replication in all who have been infected. From study of virus glycoproteins, perhaps a bit esoteric, we ended up learning something important about pathogenesis and finding new targets to limit the ability of the virus to spread.

Why did I stay in the US? Because with a good idea and a plausible way to test a hypothesis, anyone could get research support. The US was the scientific mecca for investigators wanting to test new ideas and come up with new understanding—often in unexpected ways. Is it still that mecca? Not unless we continue to support the basic research that opens up surprising new horizons and unanticipated opportunities and keeps the US at the forefront of discovery.

**Image 1:**
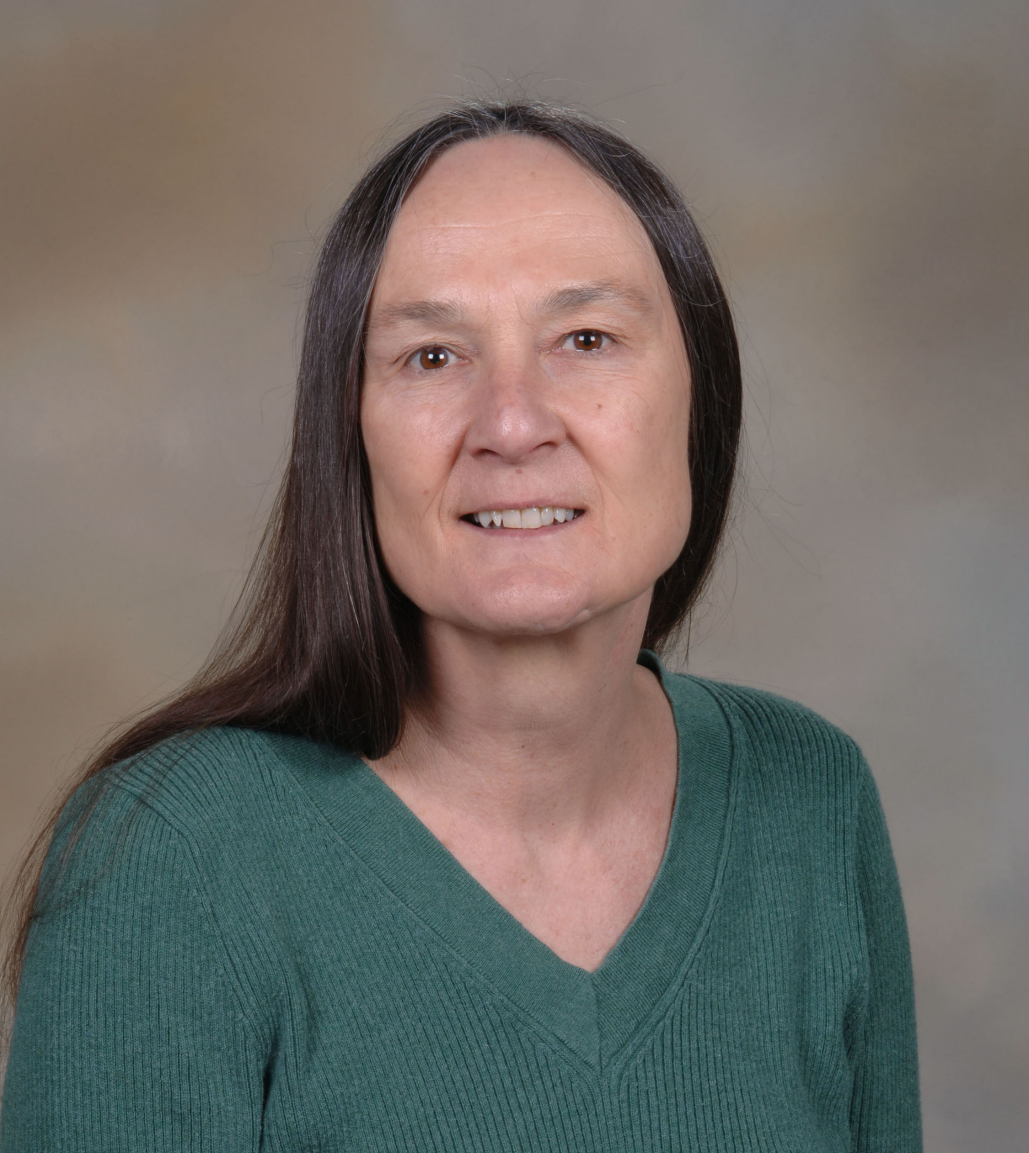
Lindsey Hutt-Fletcher.

